# In the here and now: Future thinking and social problem-solving in depression

**DOI:** 10.1371/journal.pone.0270661

**Published:** 2022-06-30

**Authors:** Saima Noreen, Barbara Dritschel

**Affiliations:** 1 Department of Psychology, De Montfort University, Leicester, England; 2 School of Psychology and Neuroscience, University of St Andrews, St Andrews, Scotland; University of Pittsburgh, UNITED STATES

## Abstract

This research investigates whether thinking about the consequences of a problem being resolved can improve social problem-solving in clinical depression. We also explore whether impaired social problem solving is related to inhibitory control. Thirty-six depressed and 43 non-depressed participants were presented with six social problems and were asked to generate consequences for the problems being resolved or remaining unresolved. Participants were then asked to solve the problems and recall all the consequences initially generated. Participants also completed the Emotional Stroop and Flanker tasks. We found that whilst depressed participants were impaired at social problem-solving after generating unresolved consequences, they were successful at generating solutions for problems for which they previously generated resolved consequences. Depressed participants were also impaired on the Stroop task, providing support for an impaired inhibitory control account of social problem-solving. These findings advance our understanding of the mechanisms underpinning social problem-solving in depression and may contribute to the development of new therapeutic interventions to improve social-problem solving in depression.

## Introduction

Social problem-solving reflects the process through which people generate effective solutions to problems experienced in everyday life [1, 2]. Given that we frequently encounter social or interpersonal problems in everyday life, such as disagreements with friends, workplace disputes and marital conflicts, the ability to solve these problems effectively is not only important for our relationships with others, but also our psychological health and mental well-being [3, 4]. Furthermore, the ability to maintain good social relationships is also important for our psychological well-being.

Deficits in social problem-solving are a central feature of depression [1, 3, 5, 6]. Priester and Chun [7] for example, found that depressed individuals exhibit a negative orientation towards a social problem compared to non-depressed healthy individuals. Furthermore, Watkins and Baracaia [8] and Goddard, Dritschel & Burton [3] found that depressed individuals generated fewer relevant steps during problem-solving and their proposed solutions were less effective than their non-depressed counterparts.

Research also suggests that rumination, which involves individuals focusing their thoughts and behaviour on their depressive symptoms and the consequences of these symptoms [9] may be a key mechanism underlying poor social problem-solving in depression. The importance of rumination in depressive disorders has been well established [10] with rumination linked to depression maintenance, negative cognitions and enhanced accessibility of negative memories [11–13].

Research has also found that rumination impairs social problem-solving, with ruminative thinking having a detrimental impact on both problem orientation and problem-solving skill. Lyubomirsky et al. [14] had dysphoric and non-dysphoric participants complete the Means-End Problem-solving Task (MEPS, [15]). In the MEPS, participants are presented with a hypothetical social problem and a positive resolution to the problem. Participants are asked to generate a number of steps to reach the proposed solution. Lyubomirsky et al. [14] found that dysphoric individuals induced to ruminate generated fewer steps and produced fewer effective solutions on the MEPS compared to dysphoric individuals who distracted themselves from their mood and their non-dysphoric counterparts. Furthermore, they also found that dysphoric individuals who ruminated appraised their problems as overwhelming and unresolvable, thus reflecting a negative problem orientation.

It is also possible, however, that poor problem-solving contributes to the maintenance of rumination in depression. As rumination involves recurrent thinking, it can be conceptualised as an attempt to problem solve and resolve unfulfilled goals [16, 17]. Indeed, research has found that the content of rumination in depression often focuses on trying to solve personal problems [14]. Furthermore, ruminative thinking continues to persist until a goal is attained or discarded. These findings suggest that a vicious cycle can ensue. There is considerable evidence that rumination impairs effective problem-solving [12, 14], increasing the likelihood of the problem being unresolved. In turn, the lack of resolution continues to trigger and maintain further rumination [18].

Another important feature of depressive thinking is hopelessness, which is defined as the extent to which an individual is pessimistic about the future [4, 19–21]. Research has found that depressed individuals generate fewer positive future events [22] which may impair social problem-solving. Noreen, Whyte & Dritschel [23], for example, had participants engage in future thinking by presenting them with a hypothetical social problem and asking them to generate the consequences of the social problem being resolved or remaining unresolved. Participants were presented with some of the solutions and were asked to solve the problem in order to achieve the resolution described. Participants were also asked to recall all of the consequences generated. The study found that participants reporting higher levels of depression and rumination were less effective at generating solutions. Furthermore, they also found that those reporting higher levels of rumination produced fewer effective solutions for social problems that they had previously generated unresolved consequences for. Individuals scoring high in rumination also recalled more of the unresolved consequences in a subsequent memory test. Taken together, these findings suggest that negative future thinking impairs the generation of effective solutions for individuals with high rumination tendencies.

One explanation for these findings may relate to the type of thinking evoked when participants were asked to think of the consequences of the problem being resolved or unresolved. According to the concreteness theory [24], there are two types of thinking; abstract and concrete. Abstract thinking is operationalised as ‘indistinct, equivocal, unclear and aggregated’ and reflects broad overarching general memories, whilst concrete thinking is ‘distinct, situational, specific and clear’ and reflects more specific individualised memories. As rumination is characterised by increased abstract thinking and reduced concrete thinking, it is possible that encouraging high ruminating individuals to think about the consequences of a problem remaining unresolved leads to greater abstract thinking, which subsequently impairs problem-solving. This is consistent with research by Watkins & Moulds [25] who found that abstract thinking, typical of rumination, impaired social problem-solving in depression. Similarly, Goddard, Dritschel & Burton [3] found that reduced social problem-solving performance in a clinically depressed sample was associated with the retrieval of spontaneous abstract categoric memories during problem-solving.

It is also possible, however, that encouraging participants to think about the consequences of a problem being resolved would encourage more concrete thinking and improve social problem-solving. Indeed, Watkins & Moulds [25] found that by encouraging participants to self-focus more concretely (i.e., focusing on the self in more concrete terms, such as, focusing on your experience of the way you feel inside) improved social problem-solving in depression. Given that Noreen, Whyte & Dritschel [23], did not have a baseline measure of problem-solving (one where no consequences were generated) it is unclear whether generating the consequences of a problem being resolved in individuals high in rumination may actually improve social problem-solving.

This is an important issue given that ineffective problem-solving has been linked to both the aetiology and maintenance of depression, which has led to the development of depression treatments that target social problem-solving [26, 27]. These treatments have demonstrated some clinical improvements in social problem-solving [28, 29], and have been found to alleviate some of the symptoms of depression [30–32]. However, these strategies do not address ruminative thinking directly associated with information *related* to social problem-solving. Therefore, it is possible that the task developed by Noreen, Whyte & Dritschel [23] may be an effective tool to improve social problem-solving in high ruminating individuals.

It is also possible that Noreen, Whyte & Dritschel’s [23] findings may be due to impaired inhibitory control. For example, people scoring high in rumination may be unable to inhibit the negative consequences they generated earlier. Difficulties inhibiting previously generated negative consequences may subsequently affect their ability to think clearly about the steps needed to solve a problem, thus resulting in impaired social problem-solving. It has been well established that inhibition is necessary to prevent irrelevant information from entering memory and instead focusing on relevant material [33]. Indeed, research has found that individuals scoring high on measures of rumination and depression demonstrate greater difficulty in inhibiting irrelevant information [34, 35]. Joormann [36], for example, found that dysphoric participants were impaired in their ability to inhibit negative material in comparison to non-depressed controls. There were no group differences, however, for positive material. Taken together, these findings suggest that both depression and rumination are associated with poor inhibitory control.

Whilst there have been a number of studies implicating the role of rumination in impairing social problem-solving, the role of inhibiting irrelevant information has not yet been examined. Thus, a key underlying process that could potentially contribute to the relationship between depression, rumination and impaired social problem-solving is currently unknown.

The aim of the present research is to provide further insight into the mechanisms that contribute to poor social problem-solving in depression. Specifically, we investigate whether thinking about the consequences of a problem being resolved can improve social problem-solving in a clinically depressed sample relative to non-depressed controls. We also examine whether thinking about the consequences of a problem being unresolved impairs social problem-solving in a clinically depressed sample significantly more than non-depressed controls. Furthermore, we also explore whether impaired social problem-solving is related to impaired inhibitory control.

To this end, participants took part in three sessions. In the first session, participants were screened for depression using the MINI-Plus. In the second session, depressed and non-depressed participants were presented with 8 vignettes that consisted of a series of interpersonal problems using a modified version [23] of the Means-End Problem-solving Task (MEPS; [15]). Participants were asked to generate four consequences of the problem being resolved for three of the vignettes and four consequences for the problem being unresolved for another three of the vignettes. Subsequently, participants were given six of the vignettes (including two that had not previously been presented, which acted as a baseline measure of problem-solving) with their resolutions and were asked to describe the steps they would take to solve the problem in order to achieve the resolution described. Following a ten-minute distraction task, participants were presented with all of the original six vignettes and were asked to recall all of the consequences that they had previously generated.

In the third session, participants were given the Flanker task [37] and the Emotional Stroop task (adapted from Strand, Oram & Hammar, [38]) to assess inhibitory control for both emotional and non-emotional stimuli. Comparing the performance on these two tasks would allow us to assess whether poor inhibitory control is greater for emotional stimuli. For social problem-solving we predicted that depressed individuals would perform poorer than non-depressed individuals in the baseline condition and also when they generate unresolved consequences. We also predicted that depressed individuals would recall more unresolved than resolved consequences, compared to non-depressed participants. For the Emotional Stroop and Flanker Tasks, we predicted that depressed participants would show inhibitory impairments on these tasks, compared to non-depressed participants. Finally, we also predicted that there would be a relationship between social-problem solving and inhibitory control, with poorer social problem-solving abilities related to impaired inhibitory control.

## Methods

### Participants

One hundred and thirteen participants (51M & 62F; age M = 23.41; SD = 3.46) took part in the initial screening session. Participants were university students that were recruited using posters advertising the study at Goldsmiths, University of London and were reimbursed for their participation (£5 per session). Participants completed the Mini-International Neuropsychiatric Interview-Plus (MINI-Plus; [39]) and the Beck Depression Inventory-II (BDI-II; [40]) in order to identify the depressed and non-depressed control groups. To be included in the depressed group, participants had to meet the criteria for current depression according to the MINI-Plus and have a minimum BDI-II score of 15. Eligibility for the controls required having no current or past Axis One disorders (e.g., anxiety disorders, dissociative disorders, mood disorders, psychotic disorders and substance use disorders) based on the MINI-Plus criteria and having a BDI-II score of 5 or below. These inclusion criteria resulted in a sample of 86 participants (41 White British; 23 British Asian (Pakistani, Indian or Bengali) and 22 Black British (African or Caribbean). A further 7 participants had to be excluded as they failed to complete all three study sessions. This resulted in 43 non-depressed control participants (17M, 26F; Mean age = 21.95; SD = 3.80) and 36 depressed participants (12M, 24F; Mean age = 21.06; SD = 4.41) in the final sample. For the currently depressed participants nine also met the criteria for dysthymic disorder, 11 met the criteria for panic disorder, 9 for social phobia, 2 for anorexia, 1 bulimia and 9 had mixed depression and anxiety. Seventeen reported taking antidepressant medications in the past and 12 had a history of past depression. The MINI-Plus was administered by a trained researcher. A second trained rater scored 25% of the interviews and there was 100% agreement regarding diagnostic status. The study was approved by the Psychology Ethics Committee, Goldsmiths, University of London. All participants provided written consent before taking part in the study.

### Materials

The Beck Depression Inventory-II [40]. The BDI-II consists of 21 items that assess both psychological and physiological symptoms of depression. Participants rate the degree to which they experience each symptom over the past two weeks on a 4- point scale. The BDI-II scale has excellent psychometric properties with good internal consistency, re-test reliability and concurrent validity with other measures of depression [41]. In the present study BDI-II was found to be highly reliable (21 items; α = .97).

The Rumination Response Scale (RRS; [42]). The RRS scale consists of 22 items that assess how participants typically respond to sad or dysphoric mood. Each item is rated on a 4- point scale (with *1 = Almost never* to *4 = Almost always*). Scores range from 22–88, with higher scores indicating greater rumination. RRS has good construct validity and internal consistency [43]. In the present study RRS was found to be highly reliable (22 items; α = .95).

The Spielberger State-Trait Anxiety Inventory (STAI; [44]). STAI is comprised of two questionnaires each containing 20-items that assess dispositional and situational anxiety, respectively. Each item is rated on a 4- point scale (with, *1 = not at all* to *4 = very much*). Scores range from 20–80 on each questionnaire, with higher scores indicating increased anxiety. Research has found that STAI has good construct and concurrent validity [44, 45]. The STAI also has good internal consistency with dispositional anxiety ranging from α = .92- α = .94 and situational anxiety ranging from α = .88 - α = .93 [44, 46]. In the present study both state and trait measures were found to be highly reliable (20 items each scale; α = .96, α = .97, respectively).

### Emotional Stroop task

The Emotional Stroop task (adapted from Strand, Oram & Hammar [38]) was used to investigate emotional inhibition and attention. The task consists of lexical and visual facial stimuli in the form of an emotional word (i.e., positive or negative) being superimposed on an emotional face (i.e., happy or sad). The task is to identify the emotional valence of the word and ignore the emotion displayed on the face. Half of the trials were congruent and the other half were incongruent. Congruent trials were defined as emotional words whose semantic meaning corresponded to the emotion of the face that it was superimposed on (i.e., the word *‘depressed’* superimposed on a sad face). Incongruent trials were defined as emotional words whose semantic meaning differed from the emotion expressed on the face that it was superimposed on (i.e., the word *‘elated’* superimposed on a sad face, or the word *‘miserable’* superimposed on a happy face).

The stimulus material consisted of 10 photographic colour images of faces (5 male & 5 female; Strand, Oram & Hammer, [38]) unknown to the participants. The images were developed at the University of St Andrews [47] with the emotional expressions and valence based on the Facial Acting Coding system developed by Ekman and colleagues [48]. Forty emotional (20 positive and 20 negative) words were superimposed in black font across the nose. All of the faces were used in the experimental session, with each face appearing with 2 positive and 2 negative words. Each word was presented twice, once with a happy face and once with a sad face. Thus, in a block of 80 trials, participants saw each of the 10 faces 8 times, and each of the 40 words twice, with half of the words superimposed on happy faces and the other half superimposed on the sad faces. The block of 80 stimuli was repeated in random order two times. The second block contained the same emotional words and faces as the first block but differed in terms of the word-face combinations. In total participants were given 160 trials.

In the task participants had to report the emotional valence of the word irrespective of the valence of the facial expression. Participants were asked to press the left arrow “<” when the word was positive and right arrow “>” when the word was negative. Prior to the experimental blocks, participants completed a practice block. This was similar to the main block but differed in terms of the faces and words that were presented. The practice block consisted of emotional words (20 positive and 20 negative) being superimposed on emotionally neutral faces. The practice block consisted of 40 trials with each emotional word-face combination presented once. To determine if there were any group differences, stroop responses were scored. In the task both correct and incorrect responses were recorded and error rates for incongruent trials were analysed. Furthermore, participant’s reaction times for correct responses were also analysed. Mean reaction times for congruent and incongruent trials were calculated. In the present study, the split half reliability for the Emotional Stroop task was found to be good (α = .42).

### Flanker task [37]

In the flanker task, participants were presented with a string of 5 letters (e.g., CCHCC) and were asked to focus their attention solely on the middle letter. Participants were instructed to press the left arrow if the target letter was H or K (straight-lined stimulus) and the right arrow if the target letter was C or S (curvy-lined stimulus). The remaining letters were one of the remaining three possible letters (H, K, C or S) and were either the same type of stimuli (e.g., HHKHH; compatible) or were a different type (CCKCC; incompatible). For the task, participants must exercise inhibitory control by ignoring the irrelevant stimuli (i.e., the outlaying four letters) and instead focus on the central stimulus.

Each trial consisted of a 1000ms fixation cross followed by the presentation of the 5-letter string. Participants were given unlimited time to respond, but were told to respond as quickly and accurately as possible. Accuracy and response times were recorded. Participants were given 2 blocks of 48 experimental trials to complete. After one block, participants were given a short 2-min break. The order of the blocks was fully counterbalanced across participants. In order to learn the response keys, participants were initially given 12 practice trials to complete. The practice trials were similar to the experimental trials but participants were given accuracy feedback (i.e., correct or incorrect response) after each trial. In the present study, the split half reliability for the Flanker task was found to be adequate (α = .42).

#### Means End Problem-Solving (MEPS; [15])

We constructed a modified version of the MEPS using eight hypothetical scenarios (adapted from Noreen, Whyte & Dritschel, [23]). The scenarios consisted of hypothetical interpersonal problems that could be encountered by a student population, such as, your supervisor finding fault with your work or your housemates not doing their chores etc. The scenarios were matched on word count, openness, difficulty in solving the hypothetical problem and the number of consequences generated (see Noreen, Whyte & Dritschel [23] for more information).

Each scenario consisted of a problem and a positive resolution. During the consequence generation phase, participants were only presented with the problem and asked to generate possible consequences for the problem either being resolved or remaining unresolved. During the problem-solving phase, participants were presented with both the problem and the positive resolution and were asked to describe the steps they would take to solve the problem and reach the proposed resolution.

The number of relevant means taken to reach the proposed solution and the effectiveness of the solutions was scored by an independent coder blind to the participant’s group status. The number of relevant means was defined as the number of relevant (and detailed) steps taken to reach the proposed solution. Effectiveness was rated using a 7- point scale with 1 being not at all effective and 7 being extremely effective. Solutions to problems were considered to be effective if they maximized positive and minimized negative consequences [49]. A second coder, also blind to participant’s group status was employed to validate findings. This coder rated 30% of the proposed solutions. Inter-rater reliability was calculated through a Pearson correlation coefficient (relevant means, *r =* .*92*, *p <* .001; effectiveness, *r =* .*95*, *p <* .001). In the present study, the split half reliability for MEPs was found to be good (α = .70).

### Procedure

The study consisted of three sessions. In the first session, participants completed the MINI-Plus, BDI II, RRS and STAI. In the second session, participants were presented with six of the eight hypothetical problems. For each problem they were given 4 minutes to generate 4 possible consequences of the problem either being resolved or remaining unresolved. Consequences were defined as *“the possible long or short-term outcomes IF the scenario was [or was not] resolved”*. Participants were asked to make sure they did not attempt to solve the scenario but only list the consequences of it being resolved or remaining unresolved. For half the hypothetical scenarios, participants generated consequences for the problem being resolved and for the remaining scenarios participants generated consequences for the problem remaining unresolved. The order of scenarios was counterbalanced so that no two ‘resolved’ or ‘not resolved’ scenarios appeared together.

Participants then completed the problem-solving task which consisted of solving six of the eight problem scenarios. These consisted of 4 scenarios that participants had generated consequences for (2 resolved and 2 unresolved) and the remaining two scenarios that participants did not generate any consequences for (a baseline measure of problem-solving).

The allocation of the scenarios to the consequence generation (resolved and unresolved) and the problem-solving phase were fully counterbalanced across participants.

For each problem-scenario, participants were presented with the problem and the positive resolution and were asked to complete the missing part of the story. Participants were given four minutes to generate a solution. Participants were subsequently given a 10-minute distraction task which involved completing some math problems. Finally, participants were given a recall test for the consequences generated earlier. Participants were presented with the 6 hypothetical scenarios presented in the recall generation phase. For each scenario, participants were given four minutes to recall all of the consequences that they had generated previously (prior to the problem-solving phase). Participants were asked to recall all of the consequences as accurately as possible. Participants were asked to recall the consequences for the baseline condition followed by the unresolved consequences and then the resolved consequences.

In a third session, participants completed the executive tasks (the Emotional Stroop task and the Flanker task). The order of the executive tasks was counterbalanced. Furthermore, the order of the administration of sessions 2 & 3 were fully counterbalanced across all participants.

## Results

### Group characteristics

The depressed group scored significantly higher than the non-depressed group on the BDI, t(36.39) = 17.33, p < .001, RRS, t(70.02) = 9.13, p < .001, and state, t(73.20) = 9.86, p < .001 and trait anxiety scales t(60.34) = 12.90, p < .001. There were no differences, however, between the depressed and non-depressed groups in terms of age, t(69.62) = .96, p = .34. See Table 1.

**Table 1:** Group differences in age, depression, rumination and anxiety measures.

	Depressed Group Mean (SD)	Non-Depressed Group Mean (SD)
**Age**	21.06 (4.41)	21.95 (3.8)
**BDI-II**	27.61 (8.53)	2.74 (1.31)
**RRS**	60.69 (8.05)	38.23 (13.52)
**STAIT**	50.53 (8.97)	30.98 (8.55)
**TRAIT**	55.86 (10.23)	29.88 (7.04)

### Social problem-solving ability: Relevant means

The mean number of relevant means (i.e., steps) taken to reach the proposed solution was assessed using a 2 (group: depressed vs. non-depressed) x 3 (condition: resolved vs. not resolved consequences vs. baseline) ANOVA. We found significant main effects of group, *F* (1, 77) = 33.66, *p <* .001, *η*^*2*^ *p* = .30, and condition, *F* (2, 77) = 50.27, *p <* .001, *η*^*2*^ *p* = .40. These were qualified by a group by condition interaction, *F* (2, 77) = 22.68, *p <* .001, *η*^*2*^ *p* = .23, with the depressed group taking fewer steps than the non-depressed group in the baseline condition, *t* (61.36) = 3.32, *p* = .002, *d* = .76 and in the unresolved condition, *t* (67.54) = 7.04, *p* < .001, *d* = 1.60. There were no differences, however, in the relevant means between the depressed and non-depressed groups in the resolved condition, *t* (58.19) = 2.03, *p* = .047, *d* = .47.

Interestingly, we also found that the non-depressed group did not differ in the relevant means between the baseline condition and the resolved, *t* (42) = 1.25, *p* = .22, *d* = .24, and unresolved conditions, *t* (42) = 1.63, *p* = .11, *d* = .24. The non-depressed group, did, however, take significantly more steps in the resolved than unresolved conditions, *t* (42) = 2.36, *p* = .02, *d* = .46. The depressed group took significantly more steps in the resolved than baseline, *t* (35) = 3.47, *p* = .001, *d* = .57, and unresolved conditions, *t* (35) = 10.50, *p* < .001, *d* = 1.76. Depressed participants, however, took fewer steps in the unresolved than the baseline condition, *t* (35) = 6.29, *p* < .001, *d* = 1.12. We also investigated the effects of gender on social problem-solving, memory accuracy and on the Emotional Stroop and Flanker tasks. We did not find any significant main or interaction effects of gender on any of these variables, all p>.05.

### Effectiveness

The effectiveness of the proposed solutions was assessed using a 2 (group: depressed vs. non-depressed) x 3 (condition: resolved vs. not resolved consequences vs. baseline) ANOVA. Our analysis found main effects of group, *F* (1, 77) = 11.35, *p <* .001, *η*^*2*^ *p* = .13, and condition, *F* (2, 77) = 13.72, *p <* .001, *η*^*2*^ *p* = .15. A significant group by condition interaction was also found, *F* (2, 77) = 3.96, *p =* .02, *η*^*2*^ *p* = .05, with the depressed group less effective at generating solutions than the non-depressed group in the baseline, *t* (72.05) = 2.53, *p* = .01, *d* = .58 and the unresolved conditions, *t* (76.73) = 4.01, *p* < .001, *d* = .90. There were no differences, however in the effectiveness of solutions generated by the depressed and non-depressed groups in the resolved condition, *t* (72.73) = 1.0, *p* = .31, *d* = .23.

Subsequent analysis also found that the non-depressed group showed no significant differences in the effectiveness of solutions generated between the baseline and resolved, *t* (42) = .11, *p* = .91, *d* = .02, and unresolved conditions, *t* (42) = 1.58, *p* = .12, *d* = .30. There were also no differences in the effectiveness of solutions generated between resolved and unresolved conditions, *t* (42) = 1.32, *p* = .20, *d* = .26. The depressed group, however, were more effective at generating solutions in the resolved than baseline, *t* (35) = 2.49, *p* = .02, *d* = .39 and unresolved conditions, *t* (35) = 6.47, *p* < .001, *d* = 1.18. The depressed group was also more effective at generating solutions in the baseline than the unresolved condition, *t* (35) = 4.35, *p* < .01, *d* = .65. See Table 2.

**Table 2:** Group differences in social problem-solving.

	Depressed Group Mean (RT)	Non-Depressed Group Mean (RT)
**Rel. Means *(Resolved)***	5.75 (1.14)	6.20 (.74)
**Rel. Means *(Unresolved)***	2.34 (1.67)	5.69 (1.37)
**Rel. Means *(Baseline)***	5.0 (1.48)	5.98 (1.05)
**Effectiveness *(Resolved)***	4.79 (1.22)	5.15 (1.89)
**Effectiveness *(Unresolved)***	3.07 (1.66)	4.66 (1.87)
**Effectiveness *(Baseline)***	4.19 (1.80)	5.19 (.66)
**Consequences *(Resolved)***	43.06 (28.26)	72.67 (22.03)
**Consequences *(Unresolved)***	74. 31 (21.12)	54.07 (27.91)
**Consequences *(Baseline)***	62.50 (22.36)	63.37 (24.15)

Rel. Means = Relevant Means; Effectiveness = Effectiveness ratings for *proposed* solutions; Consequences = Recall accuracy for consequences generated.

### Memory accuracy for consequences

In order to assess recall accuracy for the consequences generated, a 2 (group: depressed vs. control) x 3 (condition: resolved vs. unresolved consequences vs. baseline) mixed design ANOVA was conducted. There were no main effects of either group, *F* (1, 77) = .94, *p =* .36, *η*^*2*^ *p* = .01 or condition, *F* (1.84, 141.65) = 1.64, *p =* .20, *η*^*2*^ *p* = .02. However, a significant group by condition interaction was found, *F* (1.84, 141.65) = 22.89, *p <* .001, *η*^*2*^ *p* = .23, which revealed that whilst the depressed group recalled significantly fewer resolved consequences than the non-depressed group, *t* (65.55) = 5.12, *p* < .001, *d* = 1.17. they recalled significantly more unresolved consequences, *t* (76.28) = 3.66, *p* < .001, *d* = .82. There was no difference, however, between depressed and non-depressed groups in their recall of baseline consequences, *t* (76.19) = .17, *p* = .87, *d* = .04.

Subsequent analyses also revealed that the depressed group recalled significantly more unresolved than resolved consequences, *t* (35) = 6.79, *p* < .001, *d* = 1.25, and baseline consequences, *t* (35) = 2.41, *p* = .02, *d* = .54. The depressed group, however, recalled significantly fewer resolved than baseline consequences, *t* (35) = 4.22, *p* < .01, *d* = .76. Conversely, the non-depressed group recalled significantly fewer unresolved than baseline consequences, *t* (42) = 2.21, *p* = .03, *d* = .36, but recalled significantly more resolved than unresolved consequences, *t* (42) = 2.84, *p* = .007, *d* = .74. There was no difference, however, between the non-depressed groups recall of resolved and baseline consequences, *t* (42) = 1.70, *p* = .10, *d* = .40. See Table 2.

### Emotional Stroop task

#### Accuracy

A 2 (group: depressed vs. control) x 2 (valence: positive vs. negative) x 2 (distractor: happy vs. sad face) mixed design ANOVA on accuracy was conducted. The results revealed main effects of valence, *F* (1, 77) = 27.60, *p* < .001, *η*^*2*^ *p* = .26, distractor, *F* (1, 77) = 5.07, *p* = .03, *η*^*2*^ *p* = .06, and group, *F* (1, 77) = 11.08, *p* = .001, *η*^*2*^ *p* = .13. These main effects were qualified by a 3-way valence by distractor by group interaction, *F* (1, 77) = 5.26, *p =* .03, *η*^*2*^ *p* = .06, with the depressed group recalling significantly fewer positive words superimposed on negative faces than the non-depressed group, *t* (50.97) = 3.48, *p* = .001, *d* = .80. There were no differences, however, between depressed and non-depressed groups in their recall for positive words superimposed on positive faces, *t* (40.65) = 2.07, *p* = .045, *d* = .48, negative words superimposed on negative faces, *t* (72.38) = .36, *p* = .72, *d* = .08 or negative words superimposed on positive faces, *t* (58.12) = 1.07, *p* = .29, *d* = .25.

### Reaction time

A 2 (group: depressed vs. control) x 2 (valence: positive vs. negative) x 2 (distractor: happy vs. sad face) mixed design ANOVA found a main effect of group, *F* (1, 77) = 24.0, *p <* .001, *η*^*2*^ *p* = .24, with the non-depressed group significantly faster at responding than the depressed group. We also found a significant valence by distractor by group interaction, *F* (1, 77) = 5.18, *p =* .03, *η*^*2*^ *p* = .06, with the non-depressed group significantly faster at responding to positive words superimposed on positive faces, *t* (61.43) = 3.44, *p* = .001, *d* = .79, positive words superimposed on negative faces, *t* (71.42) = 3.14, *p* < .01, *d* = .71, and for negative words superimposed on positive faces, *t* (68.64) = 4.65, *p* < .001, *d* = 1.06 than the depressed group. There were no significant differences in reaction times between depressed and non-depressed groups for negative words superimposed on negative faces, *t* (75.17) = 1.25, *p* = .21, *d* = .28. We also did not find a significant effect of valence, *F* (1, 77) = 3.43, *p =* .07, *η*^*2*^ *p* = .04, and distractor, *F* (1,77) = .42, *p =* .52, *η*^*2*^ *p* = .01. See Table 3.

**Table 3:** Group differences in the Stroop and Flanker tasks.

	Depressed Group Mean (RT)	Non-Depressed Group Mean (RT)
**Stroop Accuracy- Negative Word/Negative Face**	94.17 (7.20)	93.60 (6.69)
**Stroop Accuracy- Positive Word/Positive Face**	88.75 (17.62)	95.06 (5.47)
**Stroop Accuracy- Negative Word/Positive Face**	87.01 (15.0)	90.12 (9.76)
**Stroop Accuracy- Positive Word/Negative Face**	72.29 (21.73)	86.28 (11.49)
**Stroop RT- Negative Word/ Negative Face**	785.25 (114.63)	746.25 (161.07)
**Stroop RT- Positive Word/ Positive Face**	860.11 (216.23)	712.40 (152.78)
**Stroop RT- Negative Word/ Positive Face**	930.07 (188.71)	745.62 (158.61)
**Stroop RT- Positive Word/ Negative Face**	949.22 (197.46)	815.01 (178.53)
**Flanker Accuracy-Congruent**	90.16 (9.43)	92.14 (12.03)
**Flanker Accuracy-Incongruent**	84.95 (14.60)	85.02 (18.25)
**Flanker RT-Congruent**	481.50 (89.62)	473.38 (80.18)
**Flanker RT-Incongruent**	509.38 (119.46)	499.10 (74.30)

RT = Reaction Time

### Flanker task

#### Accuracy

A 2 (group: depressed vs. control) x 2 (congruency: congruent vs. incongruent) mixed design ANOVA found a main effect of congruency, *F* (1, 77) = 16.35, *p <* .001, *η*^*2*^ *p* = .18, with participants, overall, more accurate on congruent than incongruent trials. However, we did not find a significant main effect of group, *F* (1, 77) = .13, *p =* .72, *η*^*2*^ *p* = .002, nor a group by congruency interaction, *F* (1, 77) = .39, *p =* .53, *η*^*2*^ *p* = .005.

#### Reaction time

A 2 (group: depressed vs. control) x 2 (congruency: congruent vs. incongruent) mixed design ANOVA found a main effect of congruency, *F* (1, 77) = 4.47, *p =* .04, *η*^*2*^ *p* = .06. Overall participants were faster at responding to congruent than incongruent trials. However, we did not find either a significant main effect of group, *F* (1, 77) = .32, *p =* .57, *η*^*2*^ *p* = .004, or a group by congruency interaction, *F* (1, 77) = .007, *p =* .93, *η*^*2*^ *p* = .0.

### The relationship between depression, rumination and social problem-solving

In order to determine whether there was a relationship between depression, rumination and social problem-solving, we conducted Pearson correlations. Our analysis failed to find significant correlations between depression, rumination and problem-solving abilities for the non-depressed control group; all tests p > .05. However, the correlations between depression, rumination, and the social problem-solving measures of relevant means (i.e., steps) and effectiveness for the depressed group were significant. These are presented in Table 4.

**Table 4:** Pearson correlation coefficients for depressed participants between recall of consequences, social problem-solving and self-reported depression and rumination scores.

	Depression (BDI-II)	Rumination (RRS)
**Steps *(Resolved)***	-.595***	-.435**
**Steps *(Unresolved)***	-.799***	-.790***
**Steps *(Baseline)***	-.550**	-.373*
**Effectiveness *(Resolved)***	-.678***	-.649***
**Effectiveness *(Unresolved)***	-.734***	-.706***
**Effectiveness *(Baseline)***	-.653***	-.576***
**Consequences *(Resolved)***	-.594***	-.526**
**Consequences *(Unresolved)***	-.353*	-.510**
**Consequences *(Baseline)***	-.598***	-.411*

AC = Accuracy; RT = Reaction Time

* P < 0.05

** p < 0.01

*** p < 0.001.

### Regression analyses for relevant-means

Given that we found significant correlations between depression, rumination and social problem-solving ability in the depressed group, hierarchical multiple regression analyses were conducted in order to determine whether rumination and depression predicted performance on the problem-solving task.

The analysis found that in the baseline condition (i.e., when no consequences were generated) depression predicted the number of relevant means, Beta = .55, t(35) = 2.78, *p =*. 009, with a significant model explaining approx. 26% of the variance (F (2, 33) = 7.16, *p =* .003, R^*2 =*.^ 30, R^*2 Adjusted*^ = .26). Rumination, however, failed to predict the number of relevant means, Beta = .01, t(35) = .03, *p =* .98. In the resolved condition, depression was also found to predict the number of relevant means, Beta = .56, t(35) = 2.92, *p =*. 006, with a significant model explaining approx. 32% of the variance (F (2, 33) = 9.11, *p =* .001, R^*2 =*.^ 36, R^*2 Adjusted*^ = .32). Rumination, however, again failed to predict the number of relevant means, Beta = .05, t(35) = .27, *p =* .79. In the unresolved condition, we found that both depression and rumination predicted the number of relevant means, (depression, Beta = .49, t(35) = 4.08, *p<*. 001; rumination, Beta = .46, t(35) = 3.83, *p =* .001). A significant model found that both depression and rumination explained approx. 74% of the variance (F (2, 33) = 49.57, *p<* .001, R^*2 =*.^ 75, R^*2 Adjusted*^ = .74).

### Regression analyses for effectiveness of solutions

Regression analysis revealed that for the baseline condition, depression predicted the effectiveness of the proposed solutions, Beta = .49, t(35) = 2.77, *p =*. 01, with a significant model explaining approx. 43% of the variance (F (2, 33) = 13.95, *p<* .001, R^*2 =*.^ 46, R^*2 Adjusted*^ = .43). Rumination, however, failed to predict the effectiveness of solutions, Beta = .24, t(35) = 1.38, *p =* .18. For the resolved condition, it was found that both depression and rumination predicted the effectiveness of solutions (depression, Beta = .44, t(35) = 2.67, *p =*. 01; rumination, Beta = .35, t(35) = 2.12, *p =* .04). A significant model found depression and rumination explained approx. 50% of the variance (F (2, 33) = 18.16, *p<* .001, R^*2 =*^ .52, R^*2 Adjusted*^ = .50). For the unresolved condition, it was found that both depression and rumination predicted the effectiveness of the proposed solutions (depression, Beta = .47, t(35) = 3.20, *p<*. 01; rumination, Beta = .38, t(35) = 2.59, *p =* .01). A significant model found that both depression and rumination explained approx. 59% of the variance (F (2, 33) = 26.58, *p<* .001, R^*2 =*.^ 62, R^*2 Adjusted*^ = .59). Taken together, these findings suggest whilst depression predicts the effectiveness of the proposed solutions in the baseline condition, both depression and rumination predict the effectiveness of solutions in the resolved and unresolved conditions.

### Regression analyses for consequences generated

Regression analysis were also conducted for the consequences that were generated. It was found that for the baseline condition (e.g., when no problems were solved) depression predicted the number of consequences recalled, Beta = .60, t(35) = 3.11, *p<*. 01. A significant model was found to explaining approx. 32% of the variance (F (2, 33) = 9.16, *p<* .01, R^*2 =*.^ 36, R^*2 Adjusted*^ = .32). Rumination, however, failed to predict the recall of consequences, Beta = .004, t(35) = .02, *p =* .98. In the resolved condition, it was found that depression predicted the number of consequences recalled, Beta = .44, t(35) = 2.34, *p =*. 03, with a significant model explaining approx. 34% of the variance (F (2, 33) = 10.11, *p<*. 001, R^*2 =*.^ 38, R^*2 Adjusted*^ = .34). Rumination, however, failed to predict the recall of consequences, Beta = .23, t(35) = 1.20, *p =* .24. In the unresolved condition, however, we found that rumination predicted the number of consequences recalled, Beta = .510, t(35) = 2.46, *p =*. 02, with a significant model suggesting that rumination explained approx. 22% of the variance (F (2, 32) = 5.79, *p<* .01, R^*2 =*.^ 26, R^*2 Adjusted*^ = .22). Depression, however, failed to predict recall of consequences, Beta = .01, t(35) = .04, *p =* .97. Taken together, these findings suggest that whilst depression predicts the recall of baseline and resolved consequences, rumination predicts the recall of unresolved consequences.

### Emotional Stroop performance & problem-solving abilities

As depressed and non-depressed groups showed significant differences in only one condition of the Stroop task (i.e., positive word/negative face condition), we correlated depressed participants positive word/negative face accuracy & reaction times with relevant means, effectiveness ratings and recall of consequences across all three conditions: baseline, resolved and unresolved. The analysis revealed that Emotional Stroop accuracy performance was significantly positively correlated with self-reported depression and rumination, as well as with the number of means and effectiveness scores on the problem-solving task and the recall of baseline and resolved consequences. Furthermore, a negative correlation was found for the reaction times to the positive word negative face condition and self-reported depression, self-reported rumination, number of steps generated in the resolved and unresolved conditions, as well as, the effectiveness in the resolved condition. See Table 5. We also correlated non-depressed participants positive word/negative face accuracy & reaction times with relevant means, effectiveness ratings and recall of consequences across baseline, resolved and unresolved conditions. This analysis only found a significant relationship between positive word/negative face reaction times and recall of unresolved consequences, *r*(43) = -.31, *p =* .02; all other tests, *p >* .05.

**Table 5:** Pearson correlation coefficients for depressed participants between Stroop performance in the positive word/negative face condition, and recall of consequences, social problem-solving and self-reported depression and rumination.

	Stroop Pos Word/Neg Face Acc (N = 79)	Stroop Pos Word/Neg Face RT (N = 79)
**Stroop Pos Word/Neg Face Accuracy**		-.532**
**Stroop Pos Word/Neg Face RT**	-.532**	
**BDI-II**	.847***	.465**
**RRS**	-.590*	-.470**
**Steps *(Resolved)***	.653***.	-.403*
**Steps *(Unresolved)***	.661***.	-.337*
**Steps *(Baseline)***	.587***.	-.121 ns
**Effectiveness *(Resolved)***	.535**	-.515**
**Effectiveness *(Unresolved)***	.578***	-.037 ns
**Effectiveness *(Baseline)***	.532**	-.286 ns
**Consequences *(Resolved)***	.431**	-.162 ns
**Consequences *(Unresolved)***	.315 ns (p = .06)	-.122 ns
**Consequences *(Baseline)***	.601***	-.291 ns

AC = Accuracy; RT = Reaction Time

* P < 0.05

** p < 0.01

*** p < 0.001.

## Discussion

### The impact of thinking about the consequences being resolved versus unresolved on social problem-solving

The aim of the current study was to determine whether thinking about the consequences of social problems being resolved or remaining unresolved would have different effects on social problem-solving in a depressed versus non-depressed sample. To this end, we presented participants with a hypothetical problem and asked them to generate consequences of the problem being resolved and remaining unresolved. We also took a baseline measure of social problem solving (i.e., where no consequences were generated). Our study found that the depressed group, compared to the non-depressed group was less effective at generating solutions and produced fewer relevant means in the baseline and unresolved conditions. These findings are consistent with previous research demonstrating that depression has a detrimental impact on social problem-solving [3, 50]. The findings are also consistent with Noreen, Whyte & Dritschel [23] who found that generating the consequences of a problem remaining unresolved impaired social problem-solving in individuals scoring high in depression.

Interestingly, however, we found that there were no significant differences in the effectiveness of generating solutions and the number of relevant means between the depressed and non-depressed group in the resolved condition. Furthermore, we also found that depressed participants generated more relevant means and proposed more effective solutions to the problems in the resolved than baseline conditions. These findings are of clinical importance as they suggest that encouraging depressed individuals to think about the consequences of a problem being resolved prior to problem-solving enhances their ability to solve the problem. Given that research has found that positive problem orientation is an important factor for successful problem-solving [26], it is possible that thinking about consequences being resolved may naturally induce a positive problem-focused approach. Thus, this style of positive thinking may represent an effective strategy to improve social problem-solving in depression. Furthermore, the fact that depressed individuals were as able as non-depressed participants at generating effective solutions in this condition, suggests that depressed individuals may have intact social skills but, other cognitive-behavioural factors, such as excessive rumination or a negative-problem orientation may render them unable to select and implement these skills effectively.

### Examining the relative contributions of depression and rumination on social problem-solving as a function of thinking about the consequences being resolved versus unresolved

The regression analyses revealed that whilst depression predicted the number of relevant means in the baseline and resolved conditions, both depression and rumination predicted the number of relevant means in the unresolved condition. These findings are partially consistent with Noreen, Whyte & Dritschel [23] who found that depression predicted the number of relevant means in the resolved condition, but only rumination predicted the number of relevant means in the unresolved condition. One reason for the discrepancy in findings may relate to depression severity. The present study consisted of participants that met the diagnostic criteria for clinical depression, whilst Noreen, Whyte & Dritschel’s [23] study consisted of dysphoric participants scoring high on measures of self-reported depression and rumination. Thus, it may be that more severe levels of depressive symptomology result in impairing social problem-solving abilities. This is consistent with research which has found that depressed individuals are less skilful then nondepressed participants in solving interpersonal problems and report significantly more difficulties in making decisions concerning interpersonal problems [4, 51–53].

The fact that rumination predicted the number of relevant means in the unresolved but not resolved condition suggests that rumination, when triggered by negative thoughts or consequences, may represent an unsuitable problem-solving strategy in individuals with high levels of depression [54] and impair social problem-solving. This is consistent with research which suggests that although individuals believe rumination can help solve problems, i.e., by replaying the problem over in one’s mind and appraising it [55], when rumination is focused on negative thoughts, it can have a debilitating effect on social problem-solving [8] with individuals perceiving the problem as being more difficult to solve [14] and being less confident with the solutions they generate [56]. Thus, in the present study, when participants were asked to generate unresolved consequences, this may have triggered negative ruminative thoughts in the depressed group which led them to believe the problem was more difficult to solve. As a result, they took less steps to attempt to solve the problem.

The regression analyses also found that whilst depression was the only predictor for the effectiveness of the solutions generated in the baseline condition, both depression and rumination predicted the effectiveness of the solutions generated in the resolved and unresolved conditions. These findings are partially consistent with Noreen, Whyte & Dritschel [23] who found that whilst rumination predicted the effectiveness of the proposed solutions in the unresolved condition, only depression predicted the effectiveness of the solutions in the resolved condition.

One reason why rumination predicted the effectiveness of the proposed solutions in the resolved condition in this study but not Noreen, Whyte & Dritschel’s [23] study may relate to depression severity and the relationship between rumination and depressive symptoms. Research has found that rumination is associated with more severe and longer episodes of depression [57] and also predicts the onset of depressive episodes as well as their severity and duration [58–60]. It is important to mention that in Noreen, Whyte & Dritschel’s [23] study participants had moderate levels of depressive symptoms whilst in this study participants met a diagnostic criterion for depression. Therefore, it is possible that when individuals have moderate levels of depression, ruminative thinking is only triggered when negative information is presented. However, with more severe depression it is possible that both positive and negative information may trigger ruminative thinking. This is consistent with research which suggests that when currently depressed individuals recall positive memories their mood worsens [61], but when the positive memories are consistent with current view of the self then their mood improves [62]. Thus, recalling positive memories that are discrepant with current views of the self, worsens mood. It is possible that when depressed individuals think about the resolved consequences they might begin to ruminate about how positive resolution is discrepant with their current situation where they may have interpersonal difficulties. Future research should examine the self-relevancy of the problems to provide further insight on this issue.

The finding that rumination predicts the effectiveness of the solutions is consistent with a large body of research which has found that rumination hampers depressed individual’s problem orientation and problem-solving skills [14, 63]; see Nolen-Hoeksema, Wisco & Lyubomirsky [64] for a comprehensive review). Lyubomirsky & Nolen-Hoeksema [12], for example, found that by manipulating dysphoric participants response style by encouraging them to focus on their mood state impaired their ability to solve problems on the MEPS compared to dysphoric participants who were distracted from thinking about their mood state [14]. Taken together, these findings suggest that rumination may account for the deficits in social problem-solving in individuals high in depression.

The fact that our study found that depression, independent of rumination impaired social problem-solving in the unresolved condition may relate to the severity of depressive symptomology. Previous research has found that rumination, rather than depression impaired social problem-solving in individuals with high self-reported levels of depressive symptoms (Noreen, Whyte & Dritschel, [23]). Given that individuals who took part in the present study met the diagnostic criteria for clinical depression, it is possible that generating consequences for a problem remaining unresolved impairs social problem-solving in only those individuals that have more severe levels of depression. This is consistent with research which suggests that increased severity of depression is related to greater impairments in overall cognitive ability [65].

### Impact of consequence instruction on recall of consequences

We also found that depressed participants recalled significantly more consequences in the unresolved than resolved and baseline conditions. In contrast the non-depressed controls retrieved more resolved than non-resolved consequences. One reason for these findings may relate to the valence of the consequences generated. Participants generated more positive consequences of the problem being resolved and more negative consequences of the problem remaining unresolved. These findings are consistent with research on mood congruency effects which suggests that depressed individuals exhibit enhanced memory for negative material whilst healthy individuals demonstrate the opposite pattern with a memory bias for positive material ([66, 67]; see also Matt, Vazquez & Campbell, [68]) for a review of the early work in the area).

Alternatively, it is possible that depressed individuals may recall more unresolved consequences and be impaired at social problem-solving due to impaired inhibitory control. Indeed, it is possible that generating the consequences of a problem remaining unresolved encourages depressed individuals to ruminate on these consequences. As a result, they may mentally fixate on these items which subsequently impedes the generation of appropriate solutions. This is consistent with research finding that problem-solving relies on the ability to generate appropriate solutions whilst inhibiting inappropriate responses [69, 70].

### The role of inhibitory control in social problem-solving

The role of inhibitory control in impairing problem-solving is supported by the present findings. Our findings on the Emotional Stroop task revealed that depressed participants were significantly slower and less accurate at responding in the positive word/negative face condition compared to non-depressed participants. Furthermore, we also found that in the depressed group accuracy in this condition was positively correlated with the number of relevant means and the effectiveness of solutions generated on the problem-solving task, as well as self-reported rumination and depression. For response times, however, the opposite pattern of findings was observed with reaction times negatively correlated with the number of relevant means and the effectiveness of solutions generated on the problem-solving task, as well as self-reported rumination and depression. Given that the Stroop task is a measure of sustained attention and the depressed participants showed impairments in the incongruent (positive word/sad face) condition, suggests that depression is associated with an impaired ability to inhibit negative interfering information.

Interestingly, we found no effects of depression on the flanker test which was a measure of inhibitory control of non-valanced material. These findings are consistent with research which has found that both depression and rumination are associated with impairments in tasks that require inhibition of affective content [36, 71, 72]. Indeed, according to Koster, De Lissnyder, Derakshan & De Raedt [73], difficulty disengaging from negative material increases one’s susceptibility to rumination. Thus, it is possible that impaired cognitive control in depression leads to individuals ruminating on unresolved consequences which subsequently impairs problem-solving and leads to enhanced recall of the unresolved consequences.

### Clinical implications

It is important to highlight that our findings have potentially useful clinical implications. The fact that depressed participants showed no deficits at solving social problems compared non-depressed participants when resolved consequences were generated suggests that this may be an effective strategy to improve social problem-solving. Indeed, it is possible that generating resolved consequences results in a more a positive problem orientation style, which is a belief that social problems can be solved with a positive outcome. As positive problem orientation is conceptualised as an adaptive problem-solving strategy (see D’Zurilla & Nezu [26] for a review), these findings suggest that generating resolved consequences may aid social problem-solving in depression. Furthermore, the fact that positive problem orientation is significantly related to good psychological health, such as adaptive behaviour, positive mood, life satisfaction, and a higher level of subjective well-being [25], generating resolved consequences prior to problem-solving may actually help to reduce or alleviate sad mood in depression. Future research may wish to investigate the impact of generating resolved consequences on depressed participants subsequent mood and well-being in a therapeutic context. It is important to mention that there may also be other benefits of thinking about the problem being resolved prior to problem-solving. One possibility is that having a more positive problem orientation may encourage greater motivation in thinking about strategies for solving problems. Increasing motivation has been identified as an important factor for increasing engagement with coping strategies that can reduce depression [74]. Thus, it may be that focusing on thinking about the consequences of a problem being resolved positively increases motivation to engage in more active problem- solving strategies. Future research should look at changes in motivation for solving problems as a function of thinking about the consequences in depression. Another benefit of thinking about the generation of positive consequences is that it might encourage more positive goal-directed imagination. There is evidence that positive goal-directed imagination predicts well-being even after controlling for baseline levels of mental health [75]. Given that therapists often ask their clients to describe current problems, encouraging them to think about positive resolutions before they think about how to solve the problem could be important to improve not only social problem-solving specifically, but well-being more generally.

Furthermore, given that our findings suggest that poorer inhibitory control on the Stroop task is related to less effective problem solutions in the depressed group, it suggests that interventions such as mindfulness -based interventions (MBI) which influence inhibitory control might be useful for improving problem solving performance in depression. Mindfulness is a form of meditation that involves sustaining attentional focus on a chosen object (e.g., part of your body, sounds, specific thoughts or your breathing) and returning it to this anchor every time your mind starts to wander [76]. Research has found that mindfulness meditation is effective at enhancing executive control ([77–79]; for a review see Casedas, Pirrucio, Vadillo, [80]) with inhibitory control being the most consistent executive function that is improved by mindfulness mediation training [78]. With improved inhibitory control, depressed individuals may more effective at ignoring inappropriate and negative interfering thoughts from memory when trying to generate effective solutions to social-problems Future research should examine the impact of mindfulness on inhibitory control and its subsequent impact on social problem-solving.

## Limitations

It is important to mention however that the study does have some limitations. Firstly, although the study used participants that met the diagnostic criteria for clinical depression on the MINI Plus, participants were not clinically diagnosed with depression by a medical professional. Therefore, it is possible that the present findings may not be generalizable to clinically diagnosed depressed individuals. It is, however, important to mention that the MINI Plus is a structured diagnostic tool that is compatible with the diagnostic criteria of DSM-5 and is commonly used in clinical research. Furthermore, the fact that our findings of impaired social problem solving are consistent with previous studies [8] that have used clinically diagnosed depressed patients also supports the notion that our participants disorder related level of impairment is comparable to clinically depressed patients. It is also worth noting that our participants were also largely university students and therefore may not represent the general population. This is especially true of our depressed sample. By using university students, however, our depressed and non-depressed participants did not differ significantly in age or level of education, thus any differences across groups for social problem solving or inhibitory measures cannot be attributed to these factors. It is also worth noting that there are significantly higher rates of depression in university students compared to the general population [81], thus, making this population important to study.

An additional limitation concerns determining the impact of depression on social problem-solving relative to other mental disorders. There is evidence that social problem -solving is also impaired by other mental health disorders, such as, social anxiety disorder [82], eating disorders [83] and schizophrenia [84], which can co-occur with depression. In the present study we could not address this issue as we screened our participants for other psychological disorders. Therefore, the present findings cannot be attributed to the presence of any comorbid disorders. Nonetheless, future research may wish to use a larger and more clinically diverse sample size to explore the impact of comorbid disorders on social problem solving. Another limitation of the current study is that we did not ask participants whether they were currently on any psychopharmacological treatments for their depression. Indeed, it is possible that psychopharmacological treatments for depression may lead to individuals demonstrating a different pattern of findings on social problem solving and rumination. Thus, future research may wish to report whether participants are on any treatments and whether this impacts rumination and social problem solving. A final limitation is that the study was not preregistered, however it is important to note that the study predictions were based on robust previous research findings (Noreen, Whyte & Dritschel, [23]).

## Conclusion

In conclusion, our study has found that depressed participants have intact social problem-solving skills when solving problems that they have previously generated resolved consequences for. We also found that depressed participants recalled significantly more consequences in the unresolved than resolved and baseline conditions. These findings suggest that encouraging depressed individuals to think about the consequences of a problem being resolved may be an effective strategy to improve social problem-solving skills in depression. Furthermore, we also found that depressed participants had difficulty disengaging from negative interfering material on an Emotional Stroop task, providing support for an impaired inhibitory control account of social problem-solving in depression. These findings advance our understanding of social problem-solving in depression by providing a more nuanced understanding of the mechanisms underpinning social problem-solving difficulties and have implications for therapeutic interventions.
